# Mass or pace? Seasonal energy management in wintering boreal passerines

**DOI:** 10.1007/s00442-018-04332-6

**Published:** 2019-01-07

**Authors:** Juli Broggi, Johan F. Nilsson, Kari Koivula, Esa Hohtola, Jan-Åke Nilsson

**Affiliations:** 1 Section of Evolutionary Ecology, Department of Biology, University of Lund, 223 62 Lund, Sweden; 2 Estación Biológica de Doñana (CSIC), Av. Americo Vespucio 26, 41092 Seville, Spain; 3 Department of Ecology and Genetics, University of Oulu, P.O. Box 3000, 90014 Oulu, Finland

**Keywords:** Basal metabolic rate, Parus, Phenotypic integration, Winter ecology, Optimal body mass theory

## Abstract

Research on winter energy management in small vertebrates has focused on the regulation of body mass (BM) within a framework of starvation-predation trade-off. Winter-acclimatized birds exhibit a seasonal increase in both BM and basal metabolic rate (BMR), although the patterns of co-variation between the two traits remain unknown. We studied this co-variation in three different species of wild titmice, great, blue and willow tits, originating from two boreal regions at different latitudes. Seasonal change in BM and BMR was inter-dependent, particularly in the great tit; however, by contrast, no seasonal change was observed in the willow tit. BMR changed non-linearly in concert with BM with a peak in midwinter for both blue and great tits, whereas such non-linear pattern in willow tit was opposite and independent of BM. Surprisingly, BMR appears to be more sensitive to ambient temperatures than BM in all three species studied. Energy management is a multifaceted strategy that cannot be fully understood without considering reserve levels and energy expenditure simultaneously. Thus, our study indicates that the prevailing conceptual framework based on variation in BM alone is insufficient to understand seasonal energy management in small wintering passerines.

**Electronic supplementary material:**

The online version of this article (10.1007/s00442-018-04332-6) contains supplementary material, which is available to authorized users.

## Introduction

Endotherms often need to invest energy in heat production to maintain body temperature within a range compatible with life (Alexander 1999). Over-wintering at high latitudes is especially challenging for small passerines as they cannot store large internal energy reserves and, therefore, need to cover their daily energy requirements at a time when environmental conditions deteriorate, time available for feeding diminishes and non-renewable food resources become scarcer (Blem 2000). Birds track environmental conditions to prevent starvation during harsh ambient episodes (Carey and Dawson 1999), and as part of an acclimatization process raise their metabolic capacity (Broggi et al. 2004) and the level of energy reserves (Blem 1990) to meet increasing thermogenic needs. During nighttime, which is a forced fasting period, heat production is fueled by internal body stores largely composed of fat, although other internal tissues are catabolized when fat reserves are depleted (Hulbert and Else 2000).

Energy management during winter is believed to rely basically on the adaptive acquisition and storage of reserves (McNamara and Houston 1990; McNamara et al. 1994; Houston et al. 1997; Pravosudov and Grubb 1997; Brodin 2007). This scenario has been extensively studied from both a theoretical and empirical perspective, giving rise to what is known as the “optimal body mass” (OBM) theory (Lima 1986; Rogers 1987; Rogers and Smith 1993; Witter and Cuthill 1993; Houston et al. 1997). The central tenet underlying this theory is that birds should carry as large energy reserves as needed to prevent starvation risk (Krams et al. 2013) or to be able to sustain disease-induced periods of anorexia (Speakman 2018). Furthermore, since their internal reserves are rarely at its maximum, it follows that there should be some associated costs of carrying and/or gathering reserves, probably in terms of predation. Consequently, birds should optimize their energy reserves according to a predation-starvation trade-off (Lima 1986; Witter and Cuthill 1993; Gosler 1996; Bonter et al. 2013). Such predation-starvation trade-offs may vary between individuals for example according to dominance (Krams et al. 2013). Small wintering birds exhibit a pronounced daily body mass (BM) increase (Cuthill et al. 2000; MacLeod et al. 2005; Moiron et al. 2018), superimposed on a seasonal cycle known as winter fattening (Lehikoinen 1987; Rogers 1987; Haftorn 1989; Rogers and Rogers 1990; Bednekoff and Houston 1994; Koivula et al. 1995; Rintamäki et al. 2003). However, although the OBM theory has received considerable empirical support for daily patterns of BM variation (e.g., Polo et al. 2007), evidence is equivocal for energy management strategies over larger time scales and in hoarding species (e.g., Pravosudov and Grubb 1998; Brodin 2000; Broggi et al. 2003; Cooper 2007).

Furthermore, in addition to BM regulation winter-acclimatized small birds increase the capacity for metabolic output that concomitantly leads to an overall increase in the cost of maintenance, i.e., basal metabolic rate (BMR) (Kendeigh and Blem 1974; Liknes et al. 2002; Broggi et al. 2004, 2007; Swanson 2010), which is paralleled with changes in body composition (Scott et al. 1996; Liknes and Swanson 2011; Petit et al. 2014; Zheng et al. 2014). BMR is a highly plastic trait that can change in a matter of days (Piersma and Lindström 1997; Swanson and Olmstead 1999; Petit et al. 2013; Petit and Vézina 2014; Dubois et al. 2016). Still, individual birds follow consistent BMR strategies (Speakman et al. 2004) as it is a long-term repeatable trait (Broggi et al. 2009) that is heritable (Nilsson et al. 2009) and, thus, susceptible to selection (Sadowska et al. 2015). If energy is not constraining, increases in BMR have been related to increased working capacity (Nilsson 2002; Sadowska et al. 2013) and fitness (Boratynski et al. 2013; Sadowska et al. 2015), see (Biro and Stamps 2010). However, BMR it is often assumed to be shaped by indirect selection on other correlated traits, e.g., maximal metabolic rate, and thus unlikely to be adaptively modulated (Swanson et al. 2017), although evidence accumulates suggesting that BMR responds differently to environmental conditions than such correlated traits (Petit et al. 2013; Dubois et al. 2016). The ecological significance of individual variation in BMR remains poorly understood (see Burton et al. 2011), as it may change among different populations and circumstances (Rønning et al. 2015; Nilsson and Nilsson 2016).

Since both BM and BMR are phenotypically integrated traits that are highly and positively related, understanding how environmental factors affect one independently of the other requires a reciprocal standardization for a proper interpretation of each trait separately. On the one hand, BM is an emerging property of the organism, which necessarily changes with the proportion of different tissues. However, the prevalent theoretical framework assumes that winter changes in BM mostly result from variation in fat reserves (Blem 1990; Broggi 2006 and references therein). Furthermore, changes in BMR may result from variation in size of different organs or the proportion of tissues that will likely influence BM (Piersma and Lindström 1997; Ksiazek et al. 2004; Petit et al. 2014), in addition to changes in cellular aerobic metabolic intensity (Rønning et al. 2008). Absolute BM (BM_abs_) represents the whole-animal BM that includes all variation due to structural size and changes in body composition, while standardized BM by BMR (BM_std_) represents the individual BM that is independent of the variation in BMR. Variation in BMR necessarily involves variation in sizes and proportions of organs and tissues with different metabolic activity. Therefore, variation in BM_std_ would reflect variation in tissues with lower metabolic activity such as fat that largely compose the internal body reserves in birds (Klaassen and Biebach 1994). On the other hand, studies on energy metabolism have traditionally corrected BMR for BM to standardize BMR measurements across individuals and species as an estimate of metabolic intensity (Hulbert and Else 2000). However, whole-animal (BMR_abs_) and mass-specific metabolism (BMR_std_) have been recognized as different traits with different biological meaning (Hayes 2001; Nespolo and Franco 2007; Rønning et al. 2008). While BMR_abs_ represents the overall basal energy consumption of the whole animal and scales with individual size, BMR_std_ is the mass-specific BMR that reflects variation in metabolic intensity of the different organs/tissues of the individual (Rønning et al. 2008; Petit et al. 2014; Zheng et al. 2014). While mass-specific BMR has been widely used as a measure of metabolic intensity and for comparative purposes, standardizing BM for BMR is a novel approach (but see Senar et al. 2000).

Ecological research on energy management in wintering birds has traditionally focused on BM fluctuations as a proxy of reserve acquisition and storage within the starvation-predation trade-off framework (OBM), whereas changes in BMR have been considered as a byproduct of the regulation on other traits rather than a strategically modulated mechanism (e.g., Speakman et al. 2004; Burton et al. 2011). We hypothesize that BMR can be adaptively adjusted to changing environmental and ecological circumstances in addition to BM. If both traits were to be modulated in concert, albeit on different time scales (days vs. hours), the OBM would no longer be sufficient to interpret energy management in wintering small passerines. Here, we explore to what extent these two traits covary and respond independently to the environmental conditions in three species of forest dwelling passerines with distinct life-history strategies and originating from two different climatic regions to reveal potential strategies in the simultaneous regulation of BM and BMR during winter.

## Materials and methods

### Study areas and birds

We analyzed BM and BMR in 660 individuals belonging to three titmice species from two different populations, Oulu (Finland) (65°N, 25°30′E) and Lund (Sweden) (55°40′N, 13°25′E). BMR from all individuals was measured throughout the night and the next morning they were released at the point of capture. Many individuals were captured more than once and re-measured (up to six times both within and among winters) totaling 822 measurements. Free living great tits (*Parus major*), blue tits (*Cyanistes caeruleus*) and willow tits (*Poecile montanus*) were captured and measured near Oulu from winter 1999–2000 until winter 2005–2006. Birds were captured at dusk during the non-breeding season by baited funnel traps in Oulu (182 great tits, 29 blue tits and 35 willow tit individuals). Free living great and blue tits from Lund were captured after dusk while roosting in nestboxes from winter 1999–2000 until winter 2006–2007 (159 great tits and 255 blue tit individuals). All birds were captured within 2 h before and after sunset. Willow tits are absent from the Lund region and, therefore, could not be included in the regional comparison. On first capture, birds from all three species were sexed, aged and measured for biometrical variables by standard methods (Koivula and Orell 1988, Broggi et al. 2004). The different capturing methods used in the two populations have been proven innocuous and did not bias the samples obtained as shown by a subsample of individuals trapped with the alternate capture methods in each location (Broggi 2006).

Great and blue tits are temperate deciduous forest species that remain resident on their breeding grounds during winter in the southern study area (Lund). In contrast, great and blue tits in northern Finland are close to their northernmost distribution range, an area that has been colonized in historic times, and spend the winter in loose flocks near human settlements (Valkama et al. 2011). Willow tit is a boreal forest species that is well adapted to survive boreal winters without the need of human-provided food, being a highly resident forest species that hoards food within their territory that can buffer winter food shortage.

### Body mass and metabolic measurements

BMR is defined as the average minimal oxygen consumption under post-absorptive digestive conditions during the resting phase of the daily cycle of non-growing, non-reproductive animals at thermoneutrality (McNab 1997). Thus, BMR was measured as oxygen consumption during the night in an open-circuit respirometer in a dark climate chamber at a constant temperature of 25 °C, well within the thermoneutral zone for winter-acclimatized tits (own unpublished data). Outdoor air was pushed through mass-flow controllers [Oulu: initially Bronkhorst Hi-Tec F201C (Netherlands) and later FMA-A2407, Omega Engineering, Inc. (USA); Lund: Bronkhorst Hi-Tec F201C (The Netherlands)] at 300 ml/min into the metabolic chambers containing each individual. Outcoming air was scrubbed for CO_2_ and H_2_O before being directed through a multiplexer in turns of 5–10 min (depending on the respirometer) to the Oxygen analyzer [Oulu: first Servomex 1440 (UK) and later S-3A Ametek (USA); Lund: Servomex 4100 (UK)]. Each cycle lasted an hour and included reference air to control for the analyzer bias. Details on each respirometer configuration, data extraction procedures and potential sources of bias between Oulu and Lund have been described in detail elsewhere (Broggi 2006). Birds were weighed after capture at the closest 0.1 g. After the measurement night, birds were released at the point of capture.

### Statistical analyses

We used general linear mixed models to explain the seasonal variation in BM and BMR in the three species of titmice. Great tits and blue tits originated from two distinct populations differing in winter environmental conditions (Broggi et al. 2004). Hence, locality (Lund or Oulu) was included as a fixed factor in analyses involving great and blue tits. We first tested differences between populations in the relation between BM and BMR. Afterwards, we incorporated individual variables such as sex as a categorical predictor, and age (1 = 1st winter; 2 = older than 1st winter; 3 = 2nd winter, etc.) and body size (tarsus length) as continuous predictors (Senar and Pascual 1997). The environmental variables included were year of study (winter 1999–2000 = 1), date (October 1st = 1), average minimum temperature of previous month, week and day of capture (°C) (hereafter month, week and day MT). First-order interactions between locality, sex and the other variables were included in the full models. Additionally, date squared was included as a predictor variable to test for non-linear effects of season i.e., date.

Two sets of models were fitted for each dependent variable: BM and BMR. First, full models were analyzed singly, without considering BMR and BM as covariates (hereafter BM_abs_ and BMR_abs_). Second, the same full models were fitted with the incorporation of BMR as covariate in the BM model, and BM in the BMR model (hereafter BM_std_ and BMR_std,_ respectively). Models were estimated by REML and individual was included as a random factor. We used the autoregressive covariance structure and estimated DF by the Satterthwhite method. The full model was reduced in a backward step-wise manner according to the highest *p* value starting with the interactions, and AIC (Akaike’s information criterion) was used to choose when to stop the step-wise elimination process. Sequential reintroduction of each eliminated main effect back into the final model never improved the fit (as determined by AIC). Final models are presented in tables together with the corresponding AIC values, and the *F* values, DF and *p* values corresponding to each predictor. Parameter estimates ± SE are provided for continuous predictors. Full models and parameters removed are presented with the corresponding values when removed from the model (ESM). All *p* values are two-tailed. All continuous variables fulfilled the requirements of normality.

## Results

**Fig. 1:**
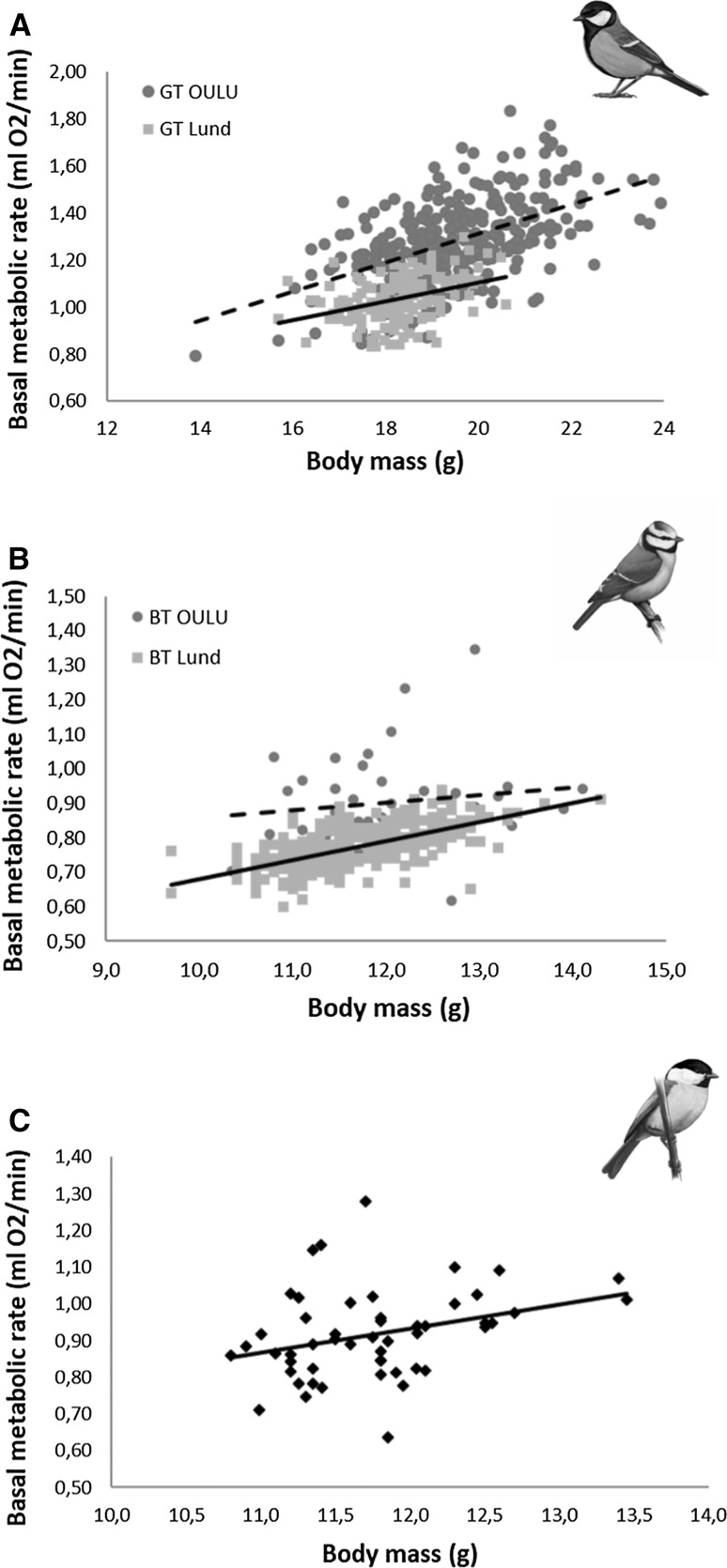
Relationship between body mass (BM_abs_, g) and winter basal metabolic rate (BMR_abs_, ml O_2_/min) with their corresponding trend lines in great tit (**a**) and blue tit (**b**) populations from Oulu (blue circles and dashed line) and Lund (red squares and solid line), and willow tits (**c**) from Oulu (black circles and solid line)

BM and BMR were positively related in all three-species studied, independently of any of the covariates considered (Fig. 1). The positive relationship was similar in the two populations of great tits (slope: Oulu 0.062 ± <0.005 vs. Lund 0.040 ± 0.012; interaction between BM and location: *F*_1,444.4_ = 2.87; *p* = 0.09; Fig. 1a), whereas blue tits in the Lund population exhibited a steeper increase in BMR for a given increase in BM than in the Oulu population (slope: Oulu 0.021 ± 0.012 vs. Lund 0.055 ± <0.005; interaction between BM and location: *F*_1,310_ = 6.39; *p* = 0.01; Fig. 1b). The slope for the willow tit in Oulu was 0.065 ± 0.029 (Fig. 1c).

### Body mass variation

**Fig. 2:**
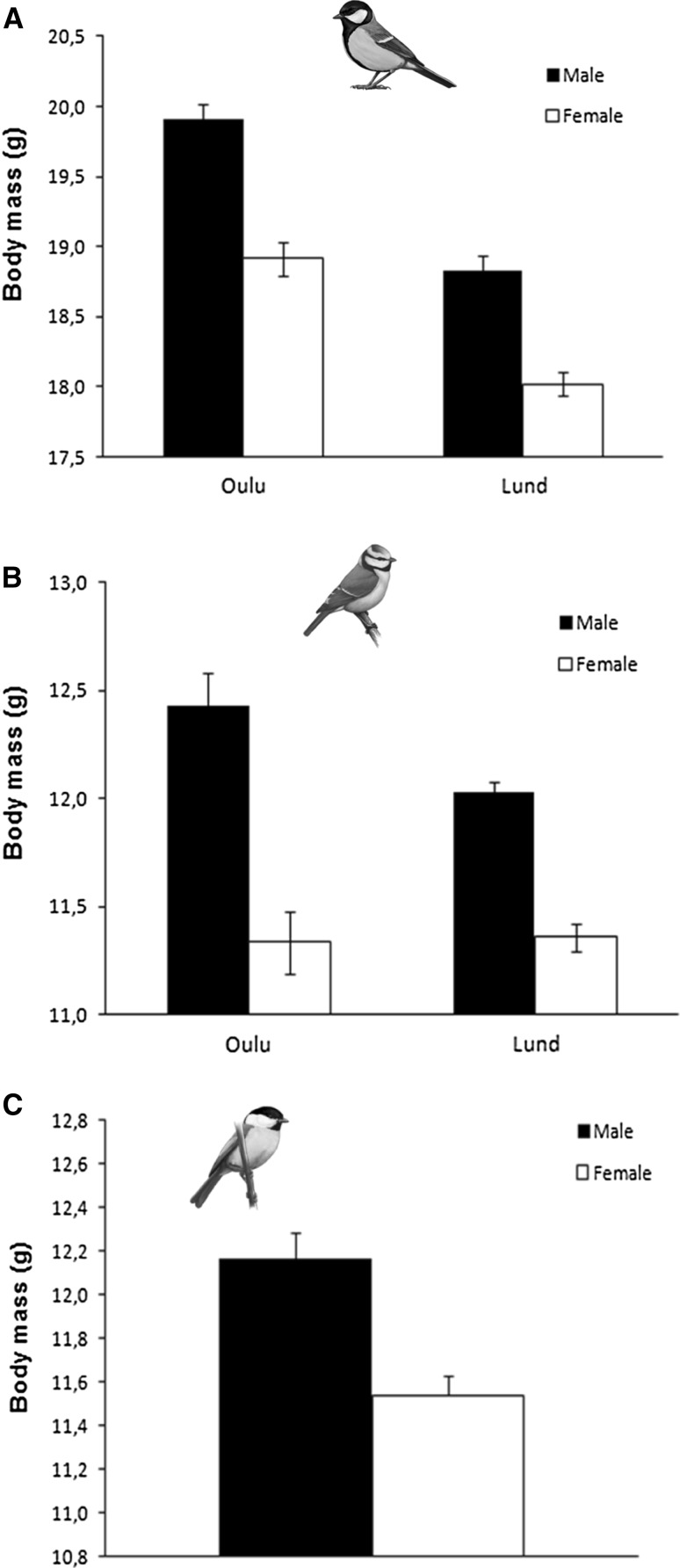
Great tit (**a**), blue tit (**b**) and willow tit (**c**) body mass between populations and sexes. Mean ± SEM are presented in black columns for males and white for females

Males in all three species were heavier than females both in BM_abs_ (Table 1 and Fig. 2) and BM_std_ (Table 1). Sexual size dimorphism in BM_abs_ varied substantially among the three species (great tit 2.48%; blue tit 6.27%; willow tit 5.45%). Furthermore, both great and blue tits were heavier in Oulu than in Lund (Table 1 and Fig. 2a, b). In both BM_abs_ and BM_std_ of blue tits, an interaction between sex and location indicates that the difference between locations was most pronounced among males (Table 1 and Fig. 2b).

**Table 1:** Results from the general linear mixed models on great tit (*Parus major*), blue tit (*Cyanistes caeruleus*) and willow tit (*Poecile montanus*) body mass (BM) as dependent variables, with (BM_std_) and without (BM_abs_) considering basal metabolic rate (BMR) as a covariate

Final models on BM considering BMR as covariate (BM_std_)																	
Great tit (*Parus major*)						Blue tit (*Cyanistes caeruleus*)						Willow tit (*Poecile montanus*)					
AIC	Predictors	*F* value	DF	*p*	Estimate ± SE	AIC	Predictors	*F* value	DF	*p*	Estimate ± SE	AIC	Predictors	*F* value	DF	*p*	Estimate ± SE
**1224.4**	**Sex**	**22.68**	**1290.7**	**< 0.001**		**473.3**	**Sex**	**47.25**	**1212.7**	**< 0.001**		**57.9**	**Sex**	**40.0**	**143.6**	**< 0.001**	
	**Loc**	**3.97**	**1145.2**	**0.048**			**Loc**	**52.12**	**1293.7**	**< 0.001**			**BMR**	**7.07**	**142.5**	**0.010**	**1.131 ± 0.425**
	**Tarsus**	**49.80**	**1306.7**	**< 0.001**	**0.488 ± 0.069**		**Tarsus**	**26.57**	**1269.9**	**< 0.001**	**0.280 ± 0.054**		**Month MT**	**8.33**	**146.6**	**0.006**	**− 0.072 ± 0.025**
	**BMR**	**13.01**	**1265.9**	**< 0.001**	**1.971 ± 0.547**		**BMR**	**63.82**	**1293.4**	**< 0.001**	**5.781 ± 0.489**						
	**Date**	**2.58**	**1254**	**0.109**	**0.007 ± 0.004**		**Date**	**4.55**	**1142.9**	**0.035**	**− 0.003 ± 0.001**						
	**Date** ^**2**^	**9.85**	**1336.4**	**0.002**	**< − 0.001 ± 0.001**		**Winter**	**10.84**	**1117.3**	**0.001**	**0.128 ± 0.039**						
	BMR*Date ^**2**^	**6.38**	**1344.4**	**0.012**	**<0.001 ± < 0.001**		Sex*Loc	**8.26**	**1209.1**	**0.005**							
	**Date** ^**2**^ *Loc	**4.65**	**1156.7**	**0.033**			BMR*Loc	**38.77**	**1290.9**	**< 0.001**							

Final models on BM without considering BMR as covariate (BM_abs_)																	
**1277.5**	**Sex**	**15.95**	**1287.8**	**< 0.001**		**593.5**	**Sex**	**34.65**	**1212.4**	**< 0.001**		**64.7**	**Sex**	**32.98**	**144.7**	**< 0.001**	
	**Loc**	**21.20**	**1,72.3**	**< 0.001**			**Loc**	**22.80**	**1185**	**< 0.001**			**Month MT**	**9.80**	**147.5**	**0.003**	**− 0.081 ± 0.026**
	**Tarsus**	**45.52**	**1308.5**	**< 0.001**	**0.530 ± 0.079**		**Tarsus**	**29.47**	**1281.9**	**< 0.001**	**0.354 ± 0.065**						
	**Date**	**9.77**	**1233.5**	**0.002**	**0.014 ± 0.005**		**Date**	**5.68**	**1103**	**0.019**	**− 0.003 ± 0.001**						
	**Date** ^**2**^	**17.12**	**1239.1**	**< 0.001**	**< − 0.001 ± < 0.001**		Winter	3.34	196.6	0.071	0.082 ± 0.045						
							**Day MT**	**5.24**	**1181.2**	**0.023**	**− 0.018 ± 0.008**						
							Sex*Loc	2.59	1204.2	0.109							

Predictors from the final models are shown, together with the corresponding AIC values, *F* values, DF and *p* values, and parameter estimates ± SE. Significant terms are presented in bold. *BMR* basal metabolic rate (mlO_2_/min), *BM* body mass (g), *Sex* gender (male = 1), *Loc* location (Oulu = 0, Lund = 1), *Tarsus* tarsus length (mm), *Date* calendar day (October 1st = 1); *Winter* winter of study (1999–2000 = 1), *MT* minimum temperature (°C)

*Interaction

**Fig. 3:**
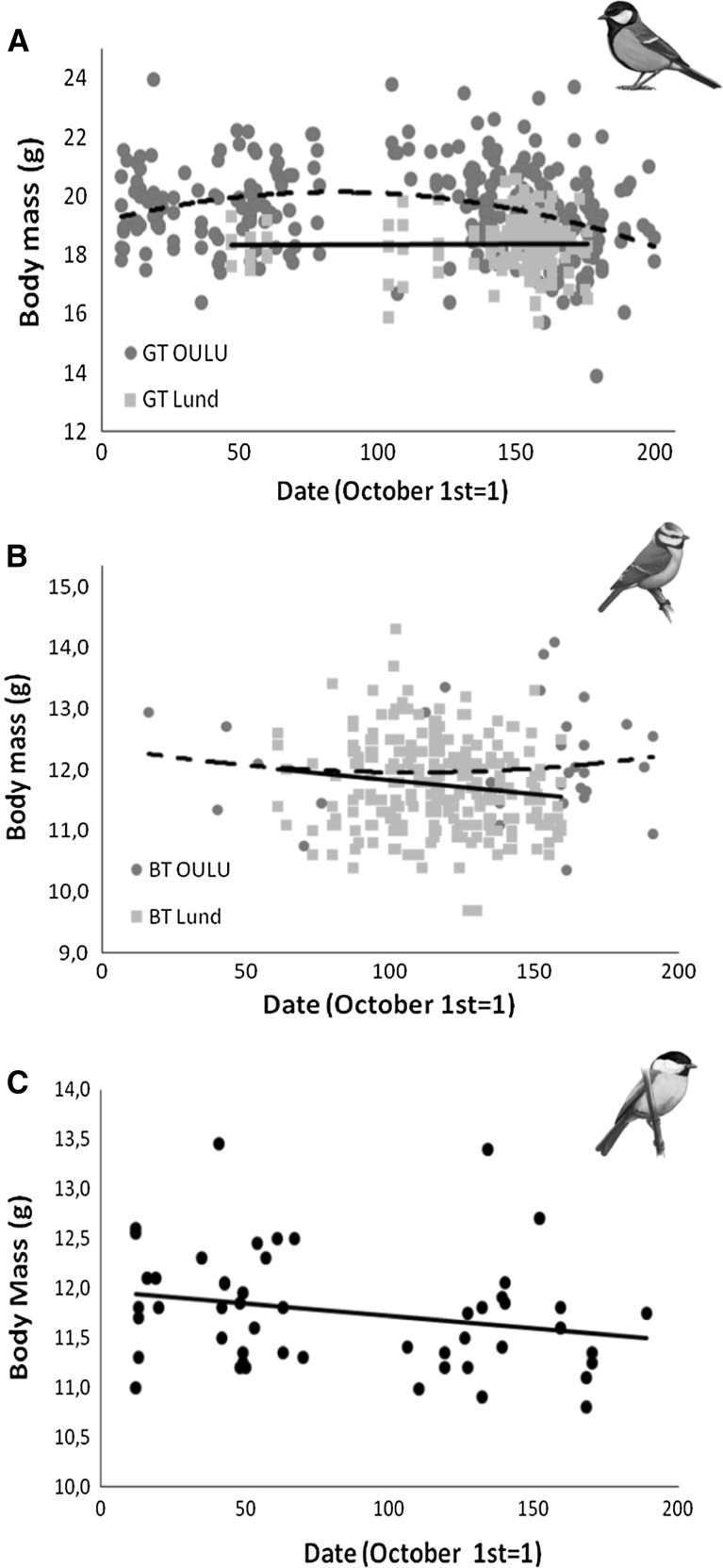
Relationship between body mass (BM_abs_, g) and date (October 1st = 1) with their corresponding tendency lines in great tit (**a**) and blue tit (**b**) populations from Oulu (blue circles and dashed line) and Lund (red squares and solid line), and willow tits (**c**) from Oulu (black circles and solid line)

There was a seasonal change in BM_abs_ in both great and blue tits, although the effect of date differed between species. While the relation was non-linear in great tits with a peak in midwinter, BM_abs_ decreased linearly through winter in blue tits (Table 1 and Fig. 3a, b). Likewise, seasonal variation in BM_std_ also differed among species. BM_std_ in great tits varied between locations throughout the season and depending on the BMR level, as shown by the interactions between date^2^ and location, and BMR, respectively. Great tit individuals with high BMR level exhibited a midwinter peak in BM_std_, which was more pronounced in Oulu than in Lund (Oulu − 1.4 × 10^−4^ ± 4.3 × 10^−5^ vs. Lund − 1.1 × 10^−4^ ± 3.9 × 10^−5^; Table 1, ESM), whereas BM_std_ linearly decreased in blue tits through winter at both locations (Table 1, ESM). Willow tits showed no significant seasonal variation in BM_abs_ nor in BM_std_ (Table 1, Fig. 3c).

Direct effects of minimum temperatures were most pronounced in willow tits, affecting both BM_abs_ and BM_std_ (Table 1), whereas in blue tits it only affected BM_abs_ and in great tits neither BM_abs_ nor BM_std_ were affected. In willow tits, both BM_abs_ and BM_std_ increased with colder monthly MT; the same relation was found in BM_abs_ for blue tits (although with daily MT). However, this relation disappeared when BMR was also accounted for (i.e., BM_std_) (Table 1). Furthermore, blue tits showed an annual increase in BM_std_ over the study period (Table 1).

### BMR variation

BMR_abs_ and BMR_std_ did not differ between sexes except in blue tits where BMR_abs_ was found to be higher in males than in females (males 0.87 ml O_2_/min ± 0.008 vs. females 0.84 ml O_2_/min ± 0.009; *t*_293_ = 10.79; *p* < 0.001). This relationship did probably stem from sexual size dimorphism, as it disappeared once BM was accounted for (Table 2). BMR_abs_ and BMR_std_ decreased in great tits with age and were higher in Oulu than in Lund for both great tits (Oulu 1.27 mlO_2_/min ± 0.010 vs. Lund 1.08 ml O_2_/min ± 0.016; *t*_393_ = 8.49; *p* < 0.001) and blue tits (Oulu 0.94 ml O_2_/min ± 0.014 vs. Lund 0.77 ml O_2_/min ± 0.005; *t*_298_ = 10.79; *p* < 0.001) (Table 2, ESM).

**Table 2:** Results from the general linear mixed models on great tit (*Parus major*), blue tit (*Cyanistes caeruleus*) and willow tit (*Poecile montanus*) basal metabolic rate (BMR) as dependent variables, with (BMR_std_) and without (BMR_abs_) considering body mass (BM) as a covariate

Final models on BMR considering BM as covariate (BMR_std_)																	
Great tit (*Parus major*)						Blue tit (*Cyanistes caeruleus*)						Willow tit (*Poecile montanus*)					
AIC	Predictors	*F* value	DF	*p*	Estimate ± SE	AIC	Predictors	*F* value	DF	*p*	Estimate ± SE	AIC	Predictors	*F* value	DF	*p*	Estimate ± SE
**− 530.3**	**Age**	**15.38**	**1378.4**	**<** **0.001**	− **0.012 ± 0.003**	− **751.6**	**Tarsus**	**4.77**	**1264.7**	**0.030**	− **0.014 ± 0.007**	− **22.8**	**BM**	**6.61**	**142.9**	**0.014**	**0.067 ± 0.026**
	**Loc**	**14.06**	**111.6**	**0.003**			**Loc**	**4.98**	**1251.8**	**0.030**			**Date**	**9.03**	**142.8**	**0.004**	− **0.005 ± 0.002**
	**BM**	**63.57**	**1444.6**	**<** **0.001**	**0.067 ± 0.008**		**BM**	**107.62**	**1290.9**	**<** **0.001**	**0.054 ± 0.005**		**Date** ^**2**^	**7.55**	**143.3**	**0.010**	**<** **0.001 ± <** **0.001**
	**Date**	**8.14**	**1379**	**0.005**	**0.002 ± <** **0.001**		**Date**	3.41	1283.3	0.066	0.002 ± < 0.001		**Winter**	**4.82**	**143.2**	**0.034**	− **0.062 ± 0.028**
	**Date** ^**2**^	1.04	1445.5	0.309	< − 0.001 ± 0.001		**Date** ^**2**^	3.76	1278.8	0.053	< 0.001 ± < 0.001		**Week MT**	**4.94**	**144.2**	**0.031**	− **0.009 ± 0.004**
	**Winter**	**9.81**	**1,86.5**	**0.002**	**0.020 ± 0.006**		**Winter**	**14.18**	**1239.4**	**<** **0.001**	− **0.019 ± 0.005**						
	**Month MT**	**7.42**	**1108.7**	**0.008**	− **0.004 ± 0.002**		**Week MT**	**7.52**	**1254.1**	**0.007**	− **0.003 ± <0.001**						
	BM*Date ^**2**^	**5.31**	**1443.8**	**0.022**	**<** − **0.001 ± <0.001**		**Date** ^**2**^ *Loc	**6.17**	**1252.3**	**0.013**							

Final models on BMR without considering BM as covariate (BMR_abs_)																	
**− 403.9**	**Age**	**5.28**	**1385.6**	**0.022**	− **0.008 ± <0.004**	− **716.0**	**Sex**	**11.11**	**1293**	**0.001**		− **23.21**	**Date**	**7.79**	**145.7**	**0.008**	− **0.005 ± 0.002**
	**Loc**	**18.98**	**125.5**	**<** **0.001**			**Loc**	**116.40**	**1298**	**<** **0.001**			**Date** ^**2**^	**5.53**	**145.3**	**0.023**	**<** **0.001 ± <** **0.001**
	**Tarsus**	**8.00**	**1410.9**	**<** **0.005**	**0.026 ± 0.009**		**Date**	**14.63**	**1306**	**<** **0.001**	**0.004 ± 0.001**		**Winter**	**6.06**	**141**	**0.018**	− **0.077 ± 0.031**
	**Date**	**11.18**	**1385.8**	**<0.001**	**0.002 ± 0.001**		**Date** ^**2**^	**17.42**	**1307**	**<0.001**	**<** − **0.001 ± <0.001**		**Week MT**	**6.82**	**145.7**	**0.012**	− **0.011 ± 0.004**
	**Date** ^**2**^	**11.57**	**1390.3**	**<** **0.001**	**<** − **0.001 ± <0.001**												
	**Month MT**	**8.67**	**1392.6**	**0.004**	− **0.006 ± 0.001**												

Predictors from the final models are shown, together with the corresponding AIC values, *F* values, DF and *p* values, and parameter estimates ± SE. Significant terms are presented in bold. *BMR* basal metabolic rate (mlO_2_/min), *BM* body mass (g), *Age* (1st year = 1), *Sex* (male = 0), *Loc* location (Oulu = 0), *Tarsus* tarsus length (mm), *Date* calendar day (October 1st = 1), *Winter* winter of study (1999–2000 = 1), *MT* minimum temperature (°C)

*Interaction

**Fig. 4:**
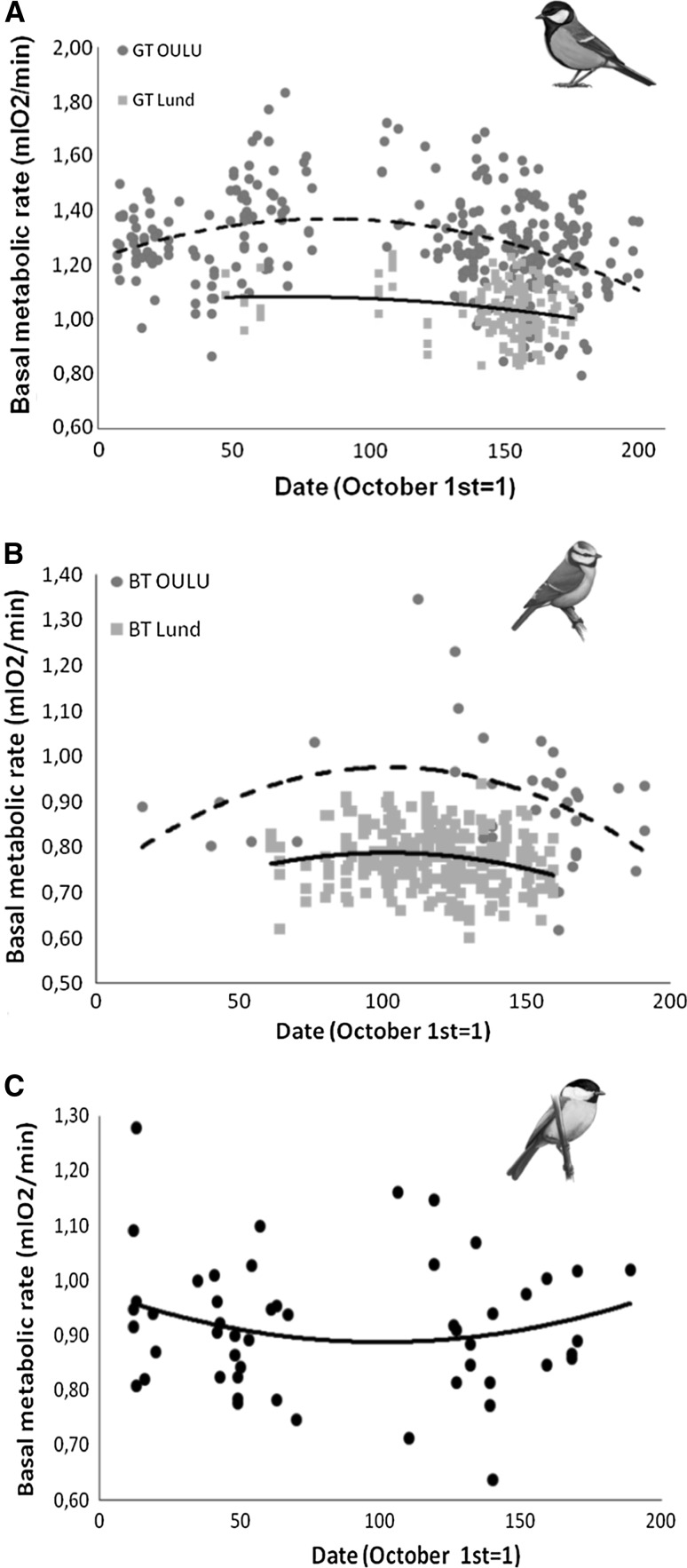
Relationship between basal metabolic rate (BMR_abs_, ml O_2_/min) and date (October 1st = 1) with their corresponding non-linear tendency lines in great tit (**a**) and blue tit (**b**) populations from Oulu (blue circles and dashed line) and Lund (red squares and solid line), and willow tits (**c**) from Oulu (black circles and solid line)

BMR_abs_ changed seasonally in all three species studied, but in different ways. In great and blue tits, this relationship was non-linear, with a peak in midwinter (Fig. 4a, b), whereas this relationship was opposite with a minimum during midwinter in willow tits (Fig. 4c). However, the seasonal pattern in BMR_std_ of great tits depended on BM, with metabolic intensity varying non-linearly when controlled for BM, as shown by a significant interaction between date^2^ and BM (Table 2, ESM). By contrast, in blue tits the seasonal increase in BMR_std_ towards midwinter was more pronounced in Oulu than in Lund (Oulu − 1.0 × 10^−5^ ± 0.4 × 10^−5^ vs. Lund − 6.8 × 10^−6^ ± 4.5 × 10^−6^), as shown by a significant interaction between date^2^ and location of origin (Table 2).

Direct effects of minimum temperatures on BMR_abs_ and BMR_std_ were apparent in all three species, with values increasing at colder temperatures. While in the great tit, variation was explained by monthly MT; shorter term averages (weekly MT) explained variation in blue and willow tits (Table 2). Furthermore, consistent annual changes in basal metabolic intensity (BMR_std_) were apparent in all three species, with values increasing over the study period for the great tit, while decreasing for blue and willow tits, whereas BMR_abs_ decreased only for willow tits over the study period (Table 2).

## Discussion

Seasonal variation in BM and BMR differs between populations and among species, and most interestingly these patterns change when BM and BMR are reciprocally standardized. BM_std_ showed a complex interactive pattern involving location, season and BMR in blue and great tits, which was not the case for BM_abs_. Analyses of BMR_std_ also resulted in interactive patterns between population, season and BM in these two species, which were not found for BMR_abs_. These results indicate altogether that sensitivity to environmental conditions and seasonality differs between standardized and absolute BM and BMR and that energy management strategies differ substantially among species.

### Location

Both absolute BM_abs_ and BMR_abs_ were higher in Oulu than in Lund for great and blue tits. This is a commonly observed latitudinal trend, in where birds exposed to colder and harsher environments react by increasing both in size and in the metabolic machinery to raise their thermogenic output (Kendeigh and Blem 1974). Furthermore, both species had higher metabolic intensity (BMR_std_) and presumably carried larger reserves (BM_std_) in Oulu than in Lund, suggesting differences in tissue proportions (lean vs. fat) and/or mitochondrial activity per gram between populations (Rønning et al. 2008; Salin et al. 2015). It should be noted that great tits and blue tits have recently colonized the Oulu region (particularly blue tits) (Valkama et al. 2011) and, during winter, rely on human-provided food sources (Orell 1989). Our results suggest that these species have adapted to withstand environmental stochasticity in energy requirements found in Oulu (Broggi et al. 2004, 2009), a strategy that may be adaptive whenever food is not limiting.

### Sex and size

Males in all three species were heavier than females, both in absolute terms and when standardized for BMR, suggesting that males were not only larger but also fatter (Table 1). This might be due to males in all three species being dominant over females (Pravosudov and Grubb 1997). Having priority of access to food, males may be able to secure more resources daily, entering the night with bigger reserves than females. Thus, we would predict a higher over-night survival in males than in females (but see Nilsson and Nilsson 2016). Although BMR_std_ did not differ between the sexes in either of the species, blue tit males had higher BMR_abs_ than females (Table 2). This difference probably stems from the more pronounced sex differences in BM and reserve levels in this species (Fig. 2), particularly in Oulu. The fact that the species has recently colonized the region (Valkama et al. 2011) and necessarily competes over very patchy (feeding tables) winter resources with the more dominant great tits could potentially explain this interpopulation pattern that certainly deserves further studies.

### Minimum temperature

Increases in BMR_std_ were found in all three species and in both localities following weekly or monthly decreasing trends in minimum temperature as commonly observed in endotherms (Alexander 1999; Blem 2000). Particularly willow tits adjusted BM_abs_ and BM_std_ in relation to perceived environmental factors, in this case monthly minimum temperatures. Similarly, blue tits were also affected by minimum temperatures, although on a more immediate time scale by increasing their BM the lower the minimum temperature on the preceding day. However, great tits did not adjust BM or BM_std_ according to the minimum temperatures, in contrast to previous studies (Gosler 2002; Krams et al. 2009; but see Krams et al. 2013). Thus, only the willow tit responded to decreasing minimum temperatures by increasing BM_std_ that could be ascribed to a change in fat reserves, whereas all three species increased BMR_std_ when the environment got colder. Interestingly, previous studies identified ambient temperatures as an ultimate rather than proximate factor explaining winter fattening, a pattern recalling the one observed in BMR (Dawson and Marsh 1986; Gosler 2002). This indicates that overall, the tits are very flexible in adjusting their metabolic phenotype to prevailing conditions, and at least on a long-time scale (months), these flexible phenotypes emphasize metabolic rather than BM adjustments. These results are in line with an increase in maximal metabolic rate and BMR (Cortés et al. 2015), as well as an increase in metabolic intensity (Liknes et al. 2014) found in black-capped chickadees (*Poecile atricapillus*) as a response to harsher environmental conditions.

### Seasonal pattern

Besides the response to prevailing conditions, the tits also followed a seasonal pattern in both BM and BMR. The great tit, which is the largest of the three species studied, was the only one exhibiting a non-linear pattern in BM with a peak in midwinter that would be in line with what is known as “true winter fattening” (Lehikoinen 1987; Rogers and Rogers 1990). Both BM_abs_ and BM_std_ followed a similar non-linear pattern, somehow more pronounced in birds from Oulu as compared to Lund. Blue tits, on the other hand, exhibited a linear decrease in BM_abs_ and BM_std_ during the season. As blue tits are subdominant in heterospecific flocks (Pravosudov and Grubb 1997), they might not be able to afford a seasonal BM strategy and may have to rely on other reserve-saving mechanisms, e.g., facultative hypothermia. Finally, willow tits did not exhibit any seasonal trend in BM at all, highlighting the fact that this boreal specialist species may have alternative wintering strategies than “winter fattening”, such as hoarding behavior (Broggi et al. 2003) and facultative nocturnal hypothermia (Reinertsen and Haftorn 1986).

BMR_abs_ exhibited a non-linear pattern of seasonal change in all three-species studied, albeit the willow tit decreased BMR_abs_ towards midwinter, opposite to the pattern found in the blue and great tits that peaked in midwinter. The same pattern was also reflected in seasonal changes in BMR_std_. Thus, BMR_std_ peaked during midwinter in great and blue tits with a more pronounced peak in Oulu than in Lund birds. Furthermore, willow tits decreased their BMR_std_ during midwinter. Thus, seasonality seems to play a more prominent role in BMR than BM variation in the three studied species. Great and blue tits respond to longer winter nights by an increased BMR_abs_ and BMR_std_, in line with common observations of increased BMR in winter (Broggi et al. 2007; Swanson 2010). Interestingly willow tits appear to engage in an opposite strategy, instead of rising expenditure to cope with increasing demands they reduce maintenance costs. Contrary to great and blue tits, this well-adapted boreal species seems to rely on hoarding behavior (Broggi 2006 and references therein), and a regular use of nighttime facultative hypothermia (Reinertsen and Haftorn 1986; Broggi et al. 2017) to withstand winter conditions at high latitudes. Thus, willow tits reduce maintenance costs and increase food predictability to cope with increasing energy demands and reduced food supply encountered during winter. Interestingly, the ecologically similar black-capped chickadee has a contrasting seasonal pattern of energy management, with midwinter peaks in both BM and BMR (Petit et al. 2013; Petit and Vézina 2014; Petit et al. 2014). This highlights the diverse energy management strategies in apparently identical ecological contexts, underlining the need for further studies to reveal the underlying mechanisms behind these differences.

### Long-term trends

The amount of reserves (BM_std_) in blue tits increased over the study years, in contrast to the other two species. Blue tits may be more sensitive to a changing climate. Because they are subdominant in heterospecific flocks, they may now, with warmer winters, be able to reach higher levels of fat reserves. Furthermore, there was a long-term trend among all three species in BMR_std_. However, while the BMR intensity in great tits increased over the studied period, it decreased in blue and willow tits. A long-term decrease in BMR intensity may reflect a selective disadvantage of having a high metabolic rate when winters become increasingly warmer, particularly for subdominant species, as previously shown in blue tits (Nilsson and Nilsson 2016). Nevertheless, interpreting sources for inter-annual variation is complicated since several factors may impinge on the individual condition during other life-history episodes that will affect future winter performance as carry-over effects (Harrison et al. 2011). Particularly, when considering pronounced physiological adjustments as those involved in changing BMR_abs_ and BMR_std_, carry-over effects are likely to be more substantial, e.g., oxidative damage and telomere shortening (Monaghan and Haussmann 2006; Isaksson et al. 2011) than those derived from internal reserve adjustments (BM_std_) (Harrison et al. 2011).

### Body mass vs. BMR

The potential for birds to manage energy expenditure according to a predation-starvation trade-off has been explored only on a theoretical basis and the focus has been on the use of facultative hypothermia (Brodin 2007) but see (Smit and McKechnie 2010). However, birds may also optimize BMR to reduce overall energy costs or to support higher energetic capacity simultaneously with management of energy reserves, albeit at different time scales (Piersma and Lindström 1997). The idea that organ-masses and energy budgets become co-optimized through natural selection is not novel (Diamond 1993). It is possible that while BM regulation may operate on a short-term scale (hours–days), by acquiring and storing of internal reserves, regulation of BMR may operate on a slightly longer time scale (days–weeks) (Piersma and Lindström 1997; Dubois et al. 2016), making it possible to increase workload capacity when food is plentiful (Nilsson 2002), or reducing overall metabolic costs under a closed energy budget (Deerenberg et al. 1998; Wiersma et al. 2005; Smit and McKechnie 2010). This variation in body composition may be due to physiological constraints (Jehl and Henry 2013) or an adaptive adjustment as found in several migratory species (Piersma and Lindström 1997).

Wintering boreal bird species are often confronted with occasional extreme food scarcity and it seems plausible that individuals can reorganize their mass accumulation strategies and energy budgets in response to new conditions. Therefore, seasonal energy management in small wintering passerines may extend beyond reserve level modulation and involve other energetic aspects as the cost of maintenance (which should reduce BMR) or support of thermogenic performance (which should increase BMR).

Ecological energetics is becoming a widely recognized field of study with relevant implications on climate-related, life-history and evolutionary studies. Although we still do not fully understand the ecological implications of changes in winter BMR, which is among the most intensively studied physiological traits (Tomlinson et al. 2014), our study suggests that it may play a prominent role in small birds’ seasonal energy management. Our results further indicate that winter fattening may not be as common a strategy in sedentary passerines at high latitudes as previously thought (Broggi et al. 2003; Cooper 2007). Therefore, when considering the response of birds to the starvation-predation trade-off over periods in where metabolic switches can take place, studies on conditions altering optimal levels of BM and BMR and how these two traits influence each other could yield fruitful outcomes.

## Electronic supplementary material

Below is the link to the electronic supplementary material.

Supplementary material 1 (DOCX 870 kb)

## Data Availability

Data will be made available from the Digital CSIC Repository 10.13140/RG.2.2.11223.52640 (Broggi et al. 2019).
