# Supplementary material for: Mass or pace? Seasonal energy management in wintering boreal passerines
Source: Oecologia. 2019 Jan 7;189(2):339–51. doi: 10.1007/s00442-018-04332-6 (PMC6394691; doi:10.1007/s00442-018-04332-6)
Supplement: Supplementary file 1 — Supplementary material 1 (DOCX 870 kb) [file 442_2018_4332_MOESM1_ESM.docx]

Electronic Supplementary Material

Figure 1 appendix. Relationship between basal metabolic rate (ml O_2_/min) standardized by body mass (BMR_std_, g) and date (October 1^st^ =1) with their corresponding non-linear tendency lines in great tit (A) and blue tit (B) populations from Oulu (blue circles and dashed line) and Lund (red squares and solid line), and willow tits (C) from Oulu (black circles and solid line).


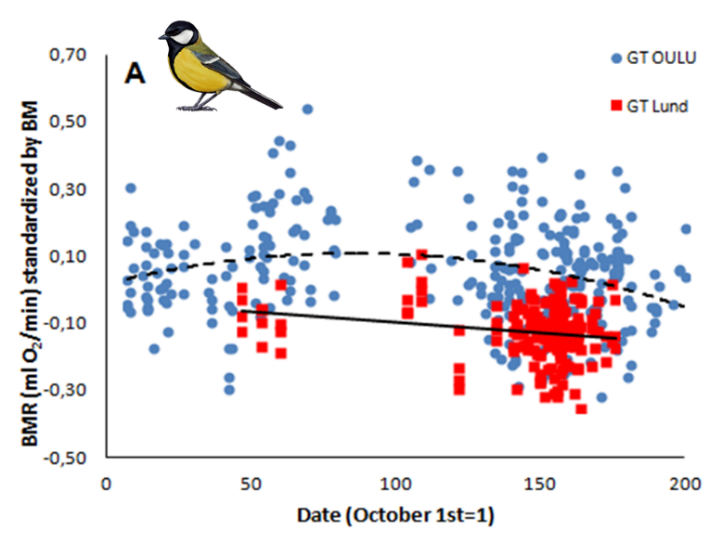

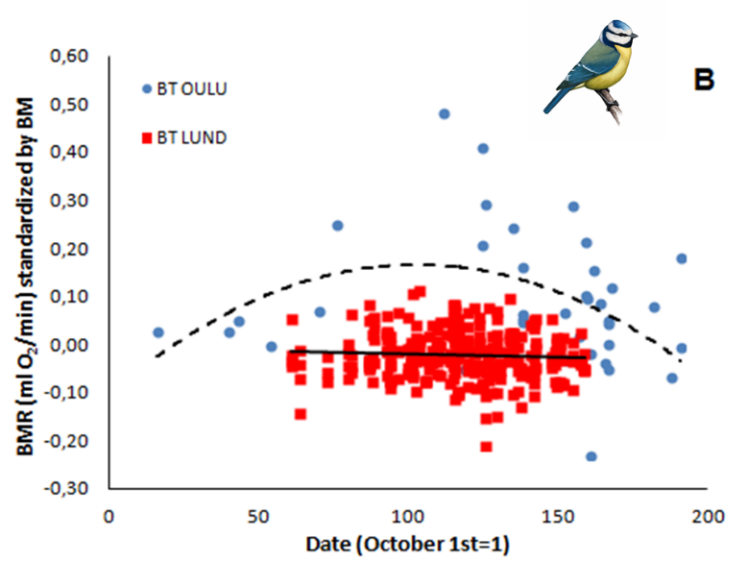

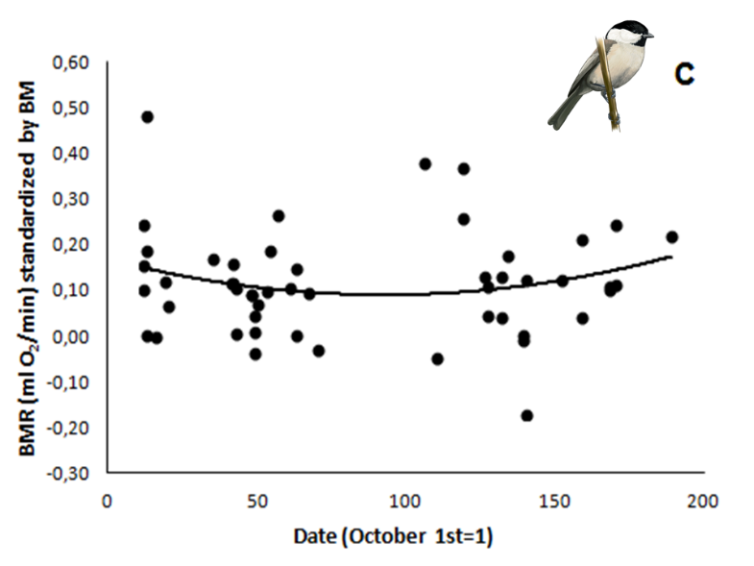


Figure 2 appendix. Relationship between body mass (BM_std_, g) standardized by basal metabolic rate (ml O_2_/min) and date (October 1^st^ =1) with their corresponding tendency lines in great tit (A) and blue tit (B) populations from Oulu (blue circles and dashed line) and Lund (red squares and solid line), and willow tits (C) from Oulu (black circles and solid line).


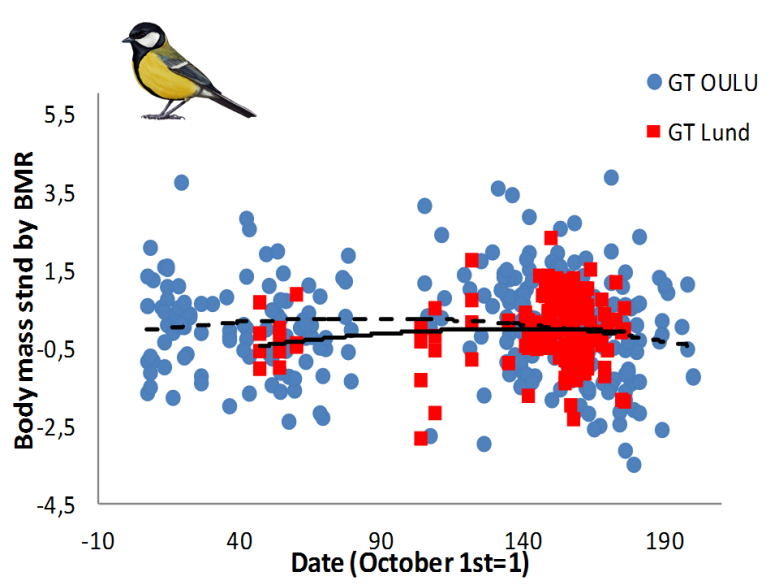

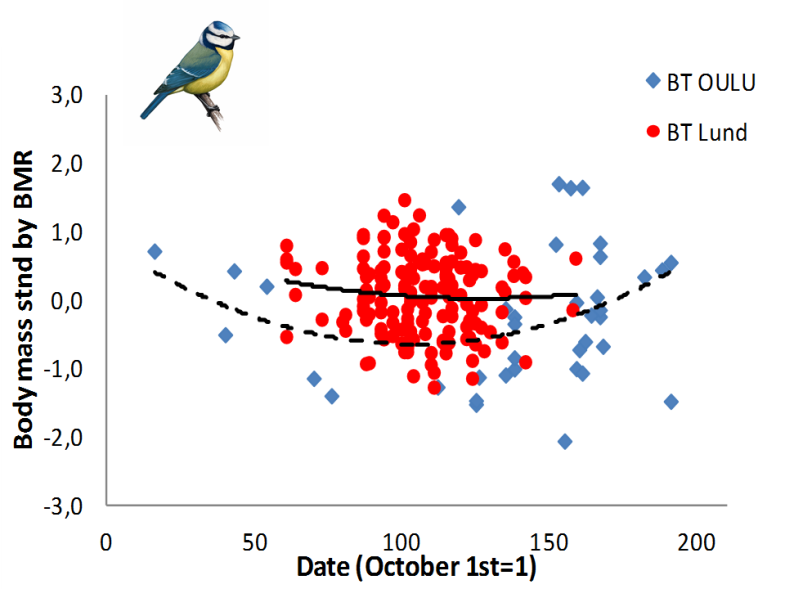

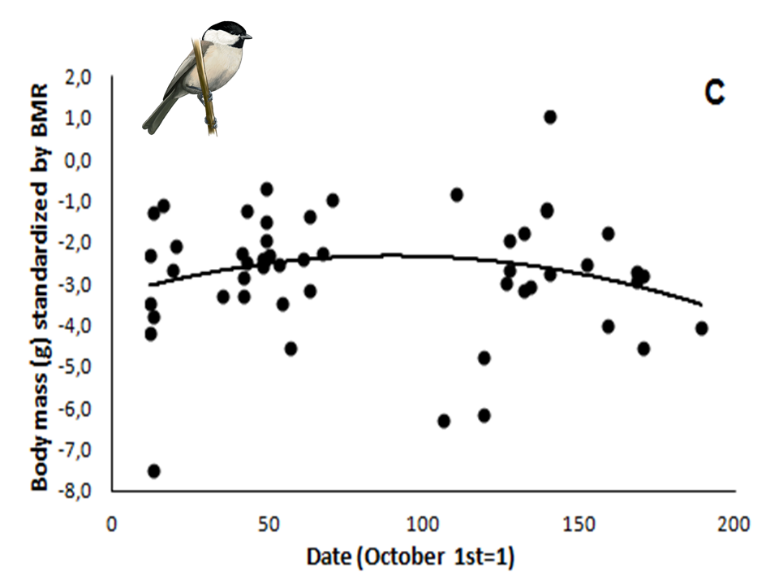


Table 1 appendix. Results from the general linear mixed model on great tit (*Parus major*) body mass as dependent variable, considering basal metabolic rate (BMR) as a covariate. All predictors are shown, together with the corresponding F values, DF and P values, and parameter estimates ± S.E. Predictors from the final model are presented in bold, and non-significant predictors are presented in order of removal from the full model with their corresponding parameters when removed from the model. AIC values are presented for the final model in bold, and for each model before the corresponding variable was removed.

| **AIC** | **Predictors** | **F value** | **DF** | **P** | **Estimate±S.E.** |
| --- | --- | --- | --- | --- | --- |
| **1224.4** | **Sex** | **22.68** | **1,290.7** | **<0.001** |  |
|  | **Location** | **3.97** | **1,145.2** | **0.048** |  |
|  | **Tarsus length (mm)** | **49.80** | **1,306.7** | **<0.001** | **0.488±0.069** |
|  | **BMR (ml O^2^/min)** | **13.01** | **1,265.9** | **<0.001** | **1.971±0.547** |
|  | Date | 2.58 | 1,254 | 0.109 | 0.007±0.004 |
|  | **Date^2^** | **9.85** | **1,336.4** | **0.002** | **<-0.001±0.001** |
|  | **BMR*Date^2^** | **6.38** | **1,344.4** | **0.012** | **<0.001±<0.001** |
|  | **Date^2^*Location** | **4.65** | **1,157.7** | **0.033** |  |
| 1228.5 | Age | 1.18 | 1,384 | 0.279 | 0.030±0.030 |
| 1234.7 | Month min. Temp. (°C) | 1.08 | 1,174.2 | 0.301 | 0.011±0.011 |
| 1238.1 | Winter | 0.81 | 1,131.9 | 0.369 | -0.044±0.049 |
| 1245.2 | Day min. Temp. (°C) | 0.06 | 1,269.8 | 0.803 | -0.002±0.008 |
| 1245.2 | Sex*Location | 1.68 | 1,326 | 0.196 |  |
| 1252.0 | Week min. Temp. (°C) | 0.01 | 1,284.8 | 0.942 | <0.001±0.012 |
| 1249.9 | BMR*Location | 0.07 | 1,372.8 | 0.786 |  |
| 1248.7 | BMR*sex | 0.08 | 1,347.4 | 0.777 |  |

Table 2 appendix. Results from the general linear mixed model on great tit (*Parus major*) body mass as dependent variable, without considering basal metabolic rate (BMR) as a covariate. All predictors are shown, together with the corresponding F values, DF and P values, and parameter estimates ± S.E. Predictors from the final model are presented in bold, and non-significant predictors are presented in order of removal from the full model with their corresponding parameters when removed from the model. AIC values are presented for the final model in bold, and for each model before the corresponding variable was removed.

| **AIC** | **Predictors** | **F value** | **DF** | | | **P** | **Estimate±S.E.** |
| --- | --- | --- | --- | --- | --- | --- | --- |
| 1278.8 | **Sex** | **15.95** | **1,287.8** | | | **<0.001** |  |
|  | **Location** | **21.20** | **1,72.3** | | | **<0.001** |  |
|  | **Tarsus length (mm)** | **45.52** | **1,308.5** | | | **<0.001** | **0.530±0.079** |
|  | **Date** | **9.77** | **1,233.5** | | | **0.002** | **0.014±0.005** |
|  | **Date^2^** | **17.12** | **1,239.1** | | | **<0.001** | **<-0.001±<0.001** |
| 1296.9 | Date^2^*Location | 2.10 | | 1,128.9 | 0.150 | |  |
| 1303.5 | Day min. Temp. (°C) | 1.16 | | 1,249.1 | 0.283 | | -0.009±0.009 |
| 1309.9 | Month min. Temp. (°C) | 0.51 | 1,183.8 | | | 0.477 | 0.009±0.013 |
| 1313.7 | Winter | 0.11 | 1,148.3 | | | 0.736 | 0.018±0.055 |
| 1318.8 | Age | 0.02 | 1,366.1 | | | 0.883 | -0.005±0.032 |
| 1325.4 | Week min.Temp. (°C) | 0.01 | 1,252.7 | | | 0.924 | 0.001±0.014 |
| 1325.8 | Sex*Location | 0.42 | 1,135 | | | 0.517 |  |

Table 3 appendix. Results from the general linear mixed model on blue tit (*Cyanistes caeruleus*) body mass as dependent variable, considering basal metabolic rate (BMR) as a covariate. All predictors are shown, together with the corresponding F values, DF and P values, and parameter estimates ± S.E. Predictors from the final model are presented in bold, and non-significant predictors are presented in order of removal from the full model with their corresponding parameters when removed from the model. AIC values are presented for the final model in bold, and for each model before the corresponding variable was removed.

| **AIC** | **Predictors** | **F value** | **DF** | **P** | **Estimate±S.E.** |
| --- | --- | --- | --- | --- | --- |
| **473.3** | **Sex** | **47.25** | **1,212.7** | **<0.001** |  |
|  | **Location** | **52.12** | **1,293.7** | **<0.001** |  |
|  | **Tarsus length (mm)** | **26.57** | **1,269.9** | **<0.001** | **0.280±0.054** |
|  | **BMR (ml O^2^/min)** | **63.82** | **1,293.4** | **<0.001** | **5.781±0.489** |
|  | **Date** | **4.55** | **1,142.9** | **0.035** | **-0.003±0.001** |
|  | **Winter** | **10.84** | **1,117.3** | **0.001** | **0.128±0.039** |
|  | **Sex*Location** | **8.26** | **1,209.1** | **0.005** |  |
|  | **BMR*Location** | **38.77** | **1,290.9** | **<0.001** |  |
| 479.6 | Day min. Temp. (°C) | 1.95 | 1,206.9 | 0.164 | -0.009±0.007 |
| 484.3 | Month min. Temp. (°C) | 2.11 | 1,243.3 | 0.147 | 0.020±0.014 |
| 489.3 | Age | 1.41 | 1,249.9 | 0.237 | 0.019±0.016 |
| 496.0 | Week min. Temp. (°C) | 0.44 | 1,168.7 | 0.507 | 0.008±0.011 |
| 514.6 | Date^2^ | 0.02 | 1,230.1 | 0.883 | <-0.001±<0.001 |
| 534.2 | Date^2^*Location | 1.36 | 1,163.9 | 0.246 |  |
| 550.9 | BMR*Date^2^ | 0.82 | 1,210.7 | 0.366 | <-0.001±<0.001 |
| 549.3 | BMR*sex | 0.11 | 1,286.3 | 0.736 |  |

Table 4 appendix. Results from the general linear mixed model on blue tit (*Cyanistes caeruleus*) body mass as dependent variable, without considering basal metabolic rate (BMR) as a covariate. All predictors are shown, together with the corresponding F values, DF and P values, and parameter estimates ± S.E. Predictors from the final model are presented in bold, and non-significant predictors are presented in order of removal from the full model with their corresponding parameters when removed from the model. AIC values are presented for the final model in bold, and for each model before the corresponding variable was removed.

| **AIC** | **Predictors** | **F value** | **DF** | | | **P** | **Estimate±S.E.** |
| --- | --- | --- | --- | --- | --- | --- | --- |
| **593.4** | **Sex** | **34.65** | **1,212.4** | | | **<0.001** |  |
|  | **Location** | **22.80** | **1,185** | | | **<0.001** |  |
|  | **Tarsus length (mm)** | **29.47** | **1,281.9** | | | **<0.001** | **0.354±0.065** |
|  | **Date** | **5.68** | **1,103** | | | **0.019** | **-0.003±0.001** |
|  | Winter | 3.34 | 1,96.6 | | | 0.071 | 0.082±0.045 |
|  | **Day min. Temp. (°C)** | **5.24** | | **1,181.2** | **0.023** | | **-0.018±0.008** |
|  | Sex*Location | 2.59 | 1,204.2 | | | 0.109 |  |
| 597.1 | Month min. Temp. (°C) | 2.82 | 1,231.8 | | | 0.095 | 0.028±0.017 |
| 614.3 | Date^2^ | 1.16 | 1,210.5 | | | 0.282 | <-0.001±<0.001 |
| 620.2 | Age | 0.09 | 1,247.8 | | | 0.767 | 0.006±0.020 |
| 626.9 | Week min. Temp. (°C) | 0.02 | 1,147.8 | | | 0.893 | -0.002±0.014 |
| 646.2 | Date^2^*Location | 1.42 | 1,154.6 | | | 0.235 |  |

Table 5 appendix. Results from the general linear mixed model on willow tit (*Poecile montanus*) body mass as dependent variable, considering basal metabolic rate (BMR) as a covariate. All predictors are shown, together with the corresponding F values, DF and P values, and parameter estimates ± S.E. Predictors from the final model are presented in bold, and non-significant predictors are presented in order of removal from the full model with their corresponding parameters when removed from the model. AIC values are presented for the final model in bold, and for each model before the corresponding variable was removed.

| **AIC** | **Predictors** | **F value** | **DF** | **P** | **Estimate±S.E.** |
| --- | --- | --- | --- | --- | --- |
| **57.9** | **Sex** | **40.0** | **1,43.6** | **<0.001** |  |
|  | **BMR (ml O^2^/min)** | **7.07** | **1,42.5** | **0.010** | **1.131±0.425** |
|  | **Month min. Temp. (°C)** | **8.33** | **1,46.6** | **0.006** | **-0.072±0.025** |
| 67.1 | Day min. Temp. (°C) | 2.58 | 1,38 | 0.120 | 0.010±0.006 |
| 71.6 | Week min. Temp. (°C) | 2.44 | 1,24.3 | 0.131 | -0.020±0.013 |
| 81.9 | Date | 1.11 | 1,42.4 | 0.298 | -0.002±0.002 |
| 84.8 | Age | 1.47 | 1,20.6 | 0.239 | -0.055±0.045 |
| 103.1 | Date^2^ | 0.40 | 1,37.6 | 0.530 | <0.001±<0.001 |
| 105.8 | Tarsus length (mm) | 0.20 | 1,39.2 | 0.659 | 0.044±0.069 |
| 107.8 | Winter | 0.03 | 1,20.7 | 0.875 | -0.023±0.144 |
| 114.5 | BMR*Date | 0.77 | 1,33.3 | 0.387 | 0.008±0.010 |
| 124.8 | BMR*Date^2^ | 0.64 | 1,36.3 | 0.429 | <0.001±<0.001 |

Table 6 appendix. Results from the general linear mixed model on willow tit (*Poecile montanus*) body mass as dependent variable, without considering basal metabolic rate (BMR) as a covariate. All predictors are shown, together with the corresponding F values, DF and P values, and parameter estimates ± S.E. Predictors from the final model are presented in bold, and non-significant predictors are presented in order of removal from the full model with their corresponding parameters when removed from the model. AIC values are presented for the final model in bold, and for each model before the corresponding variable was removed.

| **AIC** | **Predictors** | **F value** | **DF** | **P** | **Estimate±S.E.** |
| --- | --- | --- | --- | --- | --- |
| **64.7** | **Sex** | **32.98** | **1,44.7** | **<0.001** |  |
|  | **Month min. Temp. (°C)** | **9.80** | **1,47.5** | **0.003** | **-0.081±0.026** |
| 74.7 | Day min. Temp. (°C) | 1.89 | 1,38.5 | 0.178 | 0.009±0.006 |
| 79.3 | Week min. Temp. (°C) | 2.21 | 1,25.1 | 0.149 | -0.021±0.014 |
| 88.8 | Date | 1.82 | 1,43.1 | 0.185 | -0.002±0.002 |
| 105.8 | Date^2^ | 2.00 | 1,39.8 | 0.165 | <0.001±<0.001 |
| 108.1 | Age | 2.05 | 1,20.8 | 0.168 | -0.073±0.050 |
| 109.5 | Winter | 0.53 | 1,24.3 | 0.472 | -0.107±0.147 |
| 112.1 | Tarsus length (mm) | 0.21 | 1,21.4 | 0.653 | 0.046±0.101 |
